# A study protocol to explore and implement community-based point-of-care COVID-19 testing for women who use drugs in Baltimore, Maryland: The CARE study

**DOI:** 10.1371/journal.pone.0277605

**Published:** 2022-12-21

**Authors:** Catherine Tomko, Emily Clouse, Katherine Haney, Noya Galai, Katherine Footer, Kadija Ferryman, Katherine Clegg Smith, Susan G. Sherman

**Affiliations:** 1 Department of Mental Health, Johns Hopkins University Bloomberg School of Public Health, Baltimore, Maryland, United States of America; 2 Department of Health, Behavior, and Society, Johns Hopkins University Bloomberg School of Public Health, Baltimore, Maryland, United States of America; 3 Department of Epidemiology, Johns Hopkins University Bloomberg School of Public Health, Baltimore, Maryland, United States of America; 4 Berman Institute of Bioethics, Johns Hopkins University Bloomberg School of Public Health, Baltimore, Maryland, United States of America; 5 Department of Health Policy and Management, Johns Hopkins University Bloomberg School of Public Health, Baltimore, Maryland, United States of America; GERMANY

## Abstract

Women who use drugs (WWUD) experience structural vulnerabilities (e.g., housing, food insecurities) and comorbidities that elevate their susceptibility to more severe COVID-19 symptoms or fatality compared to similarly-aged women who do not use illicit drugs. Testing is a cornerstone of effective COVID prevention, however, entrenched barriers to healthcare utilization means that WWUD may have diminished accessing to COVID testing. The CARE (COVID Action Research Engagement) study first examines predisposing and enabling factors that predict COVID testing uptake over six months (baseline, 3-, and 6-month follow-up) among a cohort of WWUD (N = 250) in Baltimore, Maryland, providing a nuanced and holistic understanding of how to meaningfully engage WWUD in COVID testing. Then, point-of-care COVID testing will be implemented on a mobile outreach van affiliated with a local community-based organization primarily serving WWUD; anonymous surveys of mobile outreach guests (N = 100) will assess feasibility and acceptability of this integrated testing. The study is grounded in the Behavioral Model for Vulnerable Populations and the Theoretical Framework of Acceptability. We hypothesize that point-of-care COVID testing integrated into a low-barrier harm reduction service, such as a mobile outreach program, will be an enabling environment for COVID testing uptake in part by reducing structural impediments to testing and will be highly feasible and acceptable to participants. Strengths, limitations, and plans for results dissemination are discussed.

## Introduction

COVID-19, the viral respiratory infection caused by the SARS-CoV-2 virus, has spread rapidly around the world, though vulnerable groups such as people who use drugs (PWUD) or people experiencing homelessness, have a heightened and disproportionate risk of infection and serious complications compared to similarly-aged peers. Further, PWUD face a higher burden of infectious and chronic diseases that elevate their susceptibility to more severe COVID symptoms or fatality compared to similarly-aged women who do not use illicit drugs [1–3]. Elevated COVID susceptibility is heightened by drug inhalation which depresses breathing causing such respiratory diseases as chronic obstructive pulmonary disease and asthma, with prevalence estimates of 20% and 18%, respectively [4, 5].

Women who use drugs (WWUD) often experience significant and entrenched structural vulnerabilities (e.g., experiencing homelessness, food insecurity) that synergistically interact with comorbidities and substance use to impact their risk of COVID-19. Structural vulnerabilities reflect the socioeconomic, cultural, and political dynamics that produce and reinforce social hierarchies and constrain an individual’s or group’s ability to attain their desired health and wellbeing [6]. Housing instability, food insecurity, and stigma stemming from cultural values related to substance use are examples of the way structural vulnerabilities manifest for WWUD, all of which may increase risk of COVID acquisition. For WWUD who are experiencing homelessness, the ability to physically distance from others and shelter in place is an unrealistic expectation. Drug acquisition and use are often social activities that may necessitate interactions with drug dealers, paying clients with whom WWUD exchange sex for money or drugs, or using drugs with others (the latter of which is a critical harm reduction measure to prevent fatal overdoses). High rates of sexual and physical violence potentiate these vulnerabilities in a uniquely gendered way that is distinct from their male drug-using counterparts [7–9]. Structural vulnerabilities, coupled with a heightened biological susceptibility to COVID, render WWUD critical for COVID prevention efforts that are tailored to the population’s needs.

Accurate, easily accessible COVID-19 testing is a cornerstone of effective COVID prevention, however, COVID-19 testing remains out of reach for many WWUD due to historic and entrenched barriers to healthcare utilization [10, 11]. Studies have documented low rates of preventative healthcare or found healthcare utilization only for urgent or severe conditions [12–14]. Similar to other low-resourced populations, meeting basic needs such as shelter, food, and safe environments are more pressing and therefore prioritized over engaging in preventative healthcare [15–17]. Reduced access to healthcare and accompanying health information is particularly detrimental to COVID prevention in WWUD given the complex, multi-level barriers to healthcare engagement. The pandemic has also presented challenges to healthcare and public health systems, requiring adaptability of treatment and prevention strategies within systems that are not easily responsive to real-time change [5]. Surging need for COVID-related healthcare has diverted providers and resources away from trusted low-barrier services such as syringe services programs and drop-in centers: 43% of syringe service programs in the U.S. reported a decrease in availability of services as resources are being diverted to address the pandemic, reducing opportunities for PWUD to engage with social services [18–22]. The prolonged impact of this service reduction, along with city-wide lockdowns, may leave WWUD without trusted providers at a time when information around COVID-19 testing and vaccination from trusted medical providers is critical.

The Andersen Behavioral Model—and subsequent Behavioral Model for Vulnerable Populations—provides a framework for understanding healthcare utilization among marginalized populations such as WWUD [23, 24]. The model posits that social determinants of health influence individual-level determinants, which in turn guides decisions around an individuals’ healthcare priorities and engagement with relevant services. The framework has three domains: 1) predisposing determinants including social factors (e.g., homelessness), individual-level factors (e.g., age, drug use, race) and health beliefs (e.g., anticipated stigma, medical mistrust, perceived COVID susceptibility); 2) enabling determinants including community-level factors that facilitate or hinder healthcare access (e.g., access to healthcare, transportation); 3) need factors (e.g., competing health priorities, perceived benefits of COVID testing).

Grounded in this framework, the CARE (COVID Action Research Engagement) study is designed to understand and reduce barriers to COVID testing among WWUD. The CARE study will provide a nuanced and holistic understanding of how to meaningfully engage WWUD in COVID testing and will then implement and evaluate COVID testing on a mobile outreach van affiliated with SPARC, a local women’s center in Baltimore primarily serving the health and social needs of WWUD.

## Materials and methods

**Fig 1:**
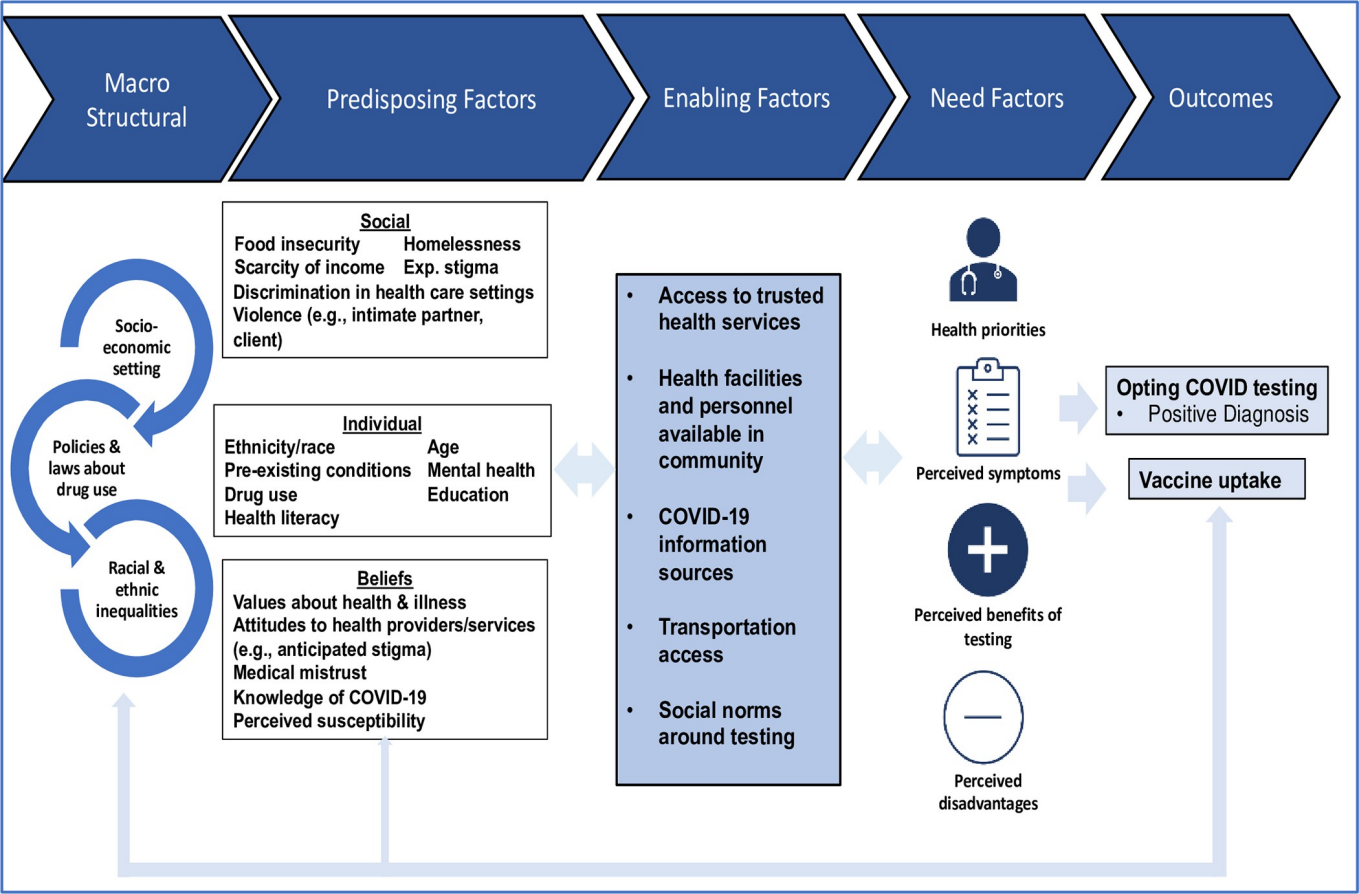
The CARE study conceptual framework.

As stated, the first phase of CARE is grounded in the Andersen Behavioral Model and subsequent expanded model adapted for application to vulnerable populations. The CARE study expands this framework (Fig 1) by positioning structural factors such as socioeconomic background and illegality of drug use as fundamental, upstream risk factors for illness and disease. These structural factors influence downstream social factors and, we posit, are dialectically-related forces in the production of individual health behaviors including COVID testing. CARE will use results gleaned from Aim 1 to implement and evaluate COVID point-of-care testing (POCT) on SPARC mobile outreach serving WWUD. The second phase is grounded in PRISM (Practical, Robust, Implementation, and Sustainability Model), a framework that emphasizes the assessment of individual- and organizational-level contextual factors that contribute to implementation outcomes [25]. The Theoretical Framework of Acceptability (TFA) [26] will guide our evaluation of POCT implementation. The TFA departs from other acceptability measures by proposing an underlying, multi-dimensional acceptability construct [26]. The TFA proposes four domains that together form the construct of acceptability: affective attitude (i.e., an individual’s feelings toward intervention participation); burden (i.e., amount of effort required to engage in intervention); self-efficacy (i.e., participant’s confidence they can engage in the behaviors required by the intervention); and perceived effectiveness (i.e., perception that the intervention will work as intended).

Specifically, the study aims to:

1. Examine predisposing and enabling factors that predict uptake of POCT over a six-month period in WWUD (N = 250) in Baltimore, Maryland.
2. Collaborate with the SPARC Center to develop and implement COVID POCT on their mobile outreach van that primarily serves WWUD in Baltimore City, Maryland.
3. Determine the feasibility and acceptability of mobile COVID POCT among WWUD (N = 100) who are clients of SPARC outreach.

We hypothesize that factors across our modified Andersen framework will contribute to WWUD uptake of COVID POCT and re-testing at follow-up and will be salient drivers of future vaccination uptake. We further hypothesize that COVID POCT integrated into a low-barrier harm reduction service, such as SPARC’s mobile outreach, will be an enabling environment for COVID testing uptake in part by reducing structural impediments to testing [19, 27, 28]. For these reasons, we hypothesize that COVID POCT on the SPARC mobile outreach van will be highly feasible and acceptable to participants.

All study activities have been approved by the Johns Hopkins University Bloomberg School of Public Health Institutional Review Board (IRB#00014847, approved 2/10/2021).

### Aim 1

Through Aim 1 we will quantitatively examine predisposing and enabling factors that are associated with COVID testing and retesting among a cohort (N = 250) of WWUD at baseline, 3-, and 6-month follow-ups. Study recruitment has begun but is not yet complete.

#### Recruitment and sampling

Participants are recruited from a mobile study van as the study team has done in recent studies of WWUD and sex workers in Baltimore City, Maryland, United States [29, 30]. Our sampling strategy uses a combination of geospatial analyses of secondary police and health data sources and primary data collection (e.g., key informant interviews with service providers and WWUD) to identify areas and times of day where WWUD congregate to develop the sampling frame. Using geocoded data from a publicly available database of 911 calls in Baltimore, we identified locations of peak drug activity based on frequency of calls mentioning drugs. After identifying the totality of locations of potential WWUD activity, we conducted windshield tours to identify which of those areas had the highest concentration of potential participants. Staff parked at identified locations and, from the vehicle, tallied the number of people observed and the number of potential participants (i.e., women that appeared to be over the age of 18) over a period of 30 minutes. Windshield tours were completed twice at each location at different times of day to detail any variations in the number of people observed.

Once the sites were determined we began recruitment, which is still ongoing. Staff park the study van at each site, discreetly approach potential participants, and describe the study. Those who are interested are then screened inside the van by a screener that masks the study’s inclusion criteria by using unrelated questions, such as arrest history. The sampled population includes WWUD who meet the following inclusion criteria: self-identify as a woman; ≥18 years; used opioids, crack cocaine, and/or powdered cocaine more than three times in the past three months.

However, recruitment has been met with challenges. At the beginning of COVID’s onset in Baltimore, the State’s Attorney’s Office implemented a temporary policy that declined to prosecute low-level, non-violent drug and sex work offenses in Baltimore (the policy was made permanent in 2021) [31]. This policy will likely prevent additional health and social burdens for the WWUD population, but it was a factor that affected our recruitment. Our sampling methods have used drug-related arrest data to aid in identifying recruitment zones; with the policy change, arrest numbers have significantly dropped, meaning that arrest data used for sampling may not be as robust as it has been previously. Further, through our windshield tours and data collection shifts we have noticed that, despite several visits to once well-known locations with WWUD, staff did not identify many potential participants on the street compared to previous studies targeting similar populations that recruited from the same locations and used the same recruitment methods. Locations that have previously yielded much higher enrollment rates have subsided substantially in this study. This change could be due to the pandemic and subsequent stay-at-home orders that have led to fewer women congregating outside as they previously had. We have attempted to remedy these recruitment challenges by engaging partners and colleagues from local community-based organizations, local businesses, and other research studies working with WWUD. For example, we have advertised the study by posting flyers in local businesses in recruitment areas (advertised as a women’s COVID testing study) and encouraged word-of-mouth referrals through social networks. We have consulted local community-based organization and other research studies serving WWUD to share experiences from the field, knowledge of changing drug markets, and to brainstorm novel methods to advertise the study.

#### Data collection

After providing written consent, all participants complete a 45–60 minute, interviewer administered survey using a tablet with programmed questionnaires. All participants are offered naloxone, condoms, fentanyl test strips, and refreshments as well as CARE swag labeled with the study logo and phone number (e.g., branded hand sanitizer). We also collect detailed locator information (e.g., phone, addresses, social media accounts) at the baseline study visit to enhance follow-up rates. This identifier information is kept separate from the study survey.

The baseline survey measures COVID testing and vaccination history and attitudes, including barriers and facilitators of both as well as history of COVID infection. The survey also measures a range of variables, including: demographics, housing, and financial status; substance use and overdose histories; sexual risk behaviors and sex work history; medical history and comorbidities; healthcare use and access; medical mistrust; and mental health. We also assess the ways in which these factors may or may not have changed as a result of COVID.

For 3- and 6-month follow-ups, we will employ methods to maintain study engagement based on our previous observational studies of mobile populations which resulted in 70%-80% follow up rates [32, 33]. We have found that our study van’s continued presence in a given community has enhanced follow-up rates, as many study participants approach the van when they think that they are due for a follow-up visit.

COVID safety precautions are followed by participants and staff on the study van. All staff members who are experiencing any COVID symptoms are required to stay home until they produce a negative test and all potential study participants must complete and pass a COVID health screening before they enter the study van. Face masks worn properly over the nose and mouth are required for research staff and study participants for the duration of the study visit. If a participant does not have a face mask of their own, or if their face mask is non-compliant per Johns Hopkins University policies (e.g., face covering with an exhalation valve, neck ‘gaiter’ coverings, or bandanas), they are provided with a surgical face mask or KN95 to wear. If a participant still does not comply with this requirement, they are not eligible to participate in the study. Participants are offered hand sanitizer and/or hand wipes upon entry and exit of the study van. Anti-viral cleaning agents are used in between participant visits to wipe down all surfaces, including tables and chairs.

#### Intervention

All study participants are offered COVID testing at the onset of their study visits, with results given in about 15 minutes. Testing is not required for study participation which will allow us to examine correlates of COVID testing. Although co-locating testing with a study visit will facilitate higher rates of testing than if women had to seek testing on their own, there are a number of potential barriers to knowing one’s COVID status (e.g., potential negative impact on drug seeking or income, medical mistrust) that we hypothesize could impede testing uptake. The study allows us to examine these barriers even with testing easily available but optional.

We use Lumira Dx SARS-CoV-2 antigen test, a 12-minute, microfluidic immunofluorescence assay for the detection of nucleocapsid protein antigen. This test has been authorized by FDA under an emergency use authorization only for the detection of SARS-CoV-2 nucleocapsid protein. When present, SARS-CoV-2 antigens bind to antibodies conjugated to particles on the test strip. These antigen-conjugate particle complexes migrate to the reaction area, where they are captured by a second set of antibodies and detected. Training for safe and precise specimen collection was provided to all study staff by a representative from Lumira DX before the start of data collection. Study staff were trained in coaching participants in swab collection, visibly demonstrating how to self-collect a specimen, taking the swab from the participant for on-site analysis, and running tests on the Lumira DX testing machines. If a participant tests positive during the study visit, the interview will immediately cease. The participant will be given a $15 gift card for their time; they are able to re-enroll in the study after two weeks.

#### Power calculation

We conducted power calculations for comparing the average rate of POCT uptake between two groups defined by a binary risk factor over 6-months of follow-up with repeated measurements. We assume that, on average, N = 200 or N = 180 participant will contribute to three data points to account for possible losses to follow-up. We further assume that the proportion of POCT in the reference category is 0.3 to 0.5, with an intra-person correlation of Rho = 0.4 and two-sided alpha = 0.05. Thus, for example, for a binary covariate with 40% in the high-risk category (e.g., injection drug use) and N = 200 we have >80% power to detect an OR = 0.5 with average of 40–50% POCT uptake in the reference category. If the total sample size is 180, with the same scenarios, power is >80% to detect OR = 0.47. For a binary factor with 70% at high risk (e.g., homeless), N = 200 and reference testing uptake rate of 40%, we have 83% power to detect an OR = 0.24. For equal group sizes (i.e., a scale cut at the median) the power is >85% to detect an OR = 1.99 and a reference testing rate of 30%. Based on these calculations we aim to recruit N = 250 to allow for losses to follow-up while preserving statistical power.

#### Statistical analysis

Through Aim 1, we will examine predisposing social factors, individual-level factors, and beliefs that are associated with COVID POCT uptake and retesting among a cohort of WWUD at baseline, 3-, and 6-month follow-up. We will start with a comprehensive descriptive analysis of all factors and outcomes, examining distributions and crude associations. To assess the association of risk factors with POCT over the 6-month follow-up period, we will apply logistic regression models adjusting for repeated measurements per person with a generalized estimating equations approach for longitudinal data. Initial models will include baseline demographic and structural factors and later models will look at time-dependent covariates such as COVID-19 testing in the prior 3-months period or vaccine uptake as well as measures of vaccine hesitancy and medical mistrust. This approach allows for maximal use of all available data. Sensitivity analyses will be performed to explore the impact of potentially differential loss to follow-up using propensity score weighting as well as evaluating longitudinal models for women that have completed all three study visits. Analyses will be performed with Stata, R, and MPlus software packages. If we have such a high rate of testing at follow-up that outcome variability is limited, we will explore other relevant outcomes such as testing positive for COVID at follow-up.

### Aim 2

In Aim 2, we will collaborate with SPARC to develop and implement an integrated COVID POCT program within their existing mobile outreach services (to begin after completing recruitment). Implementation procedures will be guided by PRISM, a framework that emphasizes the assessment of individual- and organizational-level contextual factors that contribute to implementation outcomes [25]. Outreach is conducted three to four nights per week on a mobile, retrofitted van staffed by outreach workers, most of whom have relevant lived experiences. Clients are offered safer sex (e.g., condoms, lube) and safer drug use (e.g., naloxone) supplies, basic necessities (e.g., snacks, water, clothes), personal hygiene products, referrals, and trauma-informed micro-counseling.

#### Data collection

We will conduct key informant interviews with SPARC outreach staff (N = 5), using a PRISM-informed, semi-structured interview guide focused on key characteristics of the existing program and clients, current program operations, and staff/organizational perspective on the integration of COVID POCT into SPARC outreach activities. Systematic observations (N = 6) of the outreach van will be guided by an observation form and will assess organizational and client characteristics as well as the infrastructures for POCT implementation and sustainability. Interviews will be audio-recorded and transcribed verbatim for analysis. Observations will be recorded through notes around identified topics, including client flow, space and logistics, and client-staff interactions.

#### Data analysis

We will utilize Rapid Assessment Process [34] and matrix analysis [35] to quickly analyze and synthesize key informant interviews and observation data. Coders will create templated summaries of each interview/observation using neutral domains defined *a priori* and transfer summary points into a matrix to allow for ease of identifying patterns within each domain. Results will immediately inform intervention development and implementation.

#### Training, pilot testing, and implementation

POCT will be integrated into outreach two nights per week. SPARC outreach staff trainings and implementation will be co-led by the project director and the SPARC outreach coordinator. Staff will be trained to conduct POCT and COVID risk reduction counseling, and to provide referrals for future testing and vaccinations, if requested. We will pilot the new COVID POCT for two weeks; staff will be observed during the two-week pilot to ensure competency and consistency of testing operations, which will inform any modifications to the POCT protocols.

### Aim 3

#### Recruitment and sampling

We will recruit a convenience sample (N = 100) of WWUD who engage with the SPARC van on nights that POC is offered over a period of six months. Eligibility criteria are the same as those described in Aim 1. Research study staff will stand outside the SPARC van twice a week when testing is offered to recruit, screen, and consent WWUD into the study. All women exiting the van will be approached discreetly and briefly explained the study. The survey will be anonymous to conform to SPARC outreach norms of not collecting identifying information from their clients.

#### Data collection

Once eligible women consent to study participation, they will complete a 10–15 minute, ACASI-administered survey. Consent will be oral as not to require a signature for this anonymous survey. Women will be compensated by a $10 VISA gift card. After 100 surveys are completed, we will analyze a survey question regarding prior survey engagement in order to estimate the number of repeated surveys. Based on experience, we expect collecting 250 surveys will result in 200 unique participants. The survey will include basic demographic information, prior COVID testing history and a measure of vaccine hesitancy used in Aim 1. Questions regarding study acceptability will be informed by the TFA [26]. Acceptability will be assessed using items (measured on a Likert scale) aligned with each TFA domain: affective attitude; burden; self-efficacy; and perceived effectiveness.

Participant feasibility will be assessed using survey questions that measure reasons for testing refusal. Program feasibility will be measured through SPARC secondary data, including testing uptake and comparing SPARC outreach client volume on days testing is and is not offered.

#### Statistical analysis

We first will produce a comprehensive descriptive analysis of all feasibility and acceptability variables. Among participants who received COVID tests, we will perform exploratory factor analysis to test for an underlying factor structure, as acceptability is a latent construct that the TFA proposes has several constituent domains. Using a polychoric correlation structure and principal component analysis, we will use an iterative process to determine number of factors extracted, guided by standard fitness measures (e.g., eigenvalues, scree plot, uniqueness). After deciding on a final factor structure, we will sum scores for each factor and for the full scale. Using summed scores is preferred to weighted scores when easy interpretability is highly valued and the research question is nascent, as it is here [36]. We will then conduct multivariable linear regression models with each score as the dependent variable and demographics (e.g., race) and vaccine hesitancy as predictors. We expect >50% testing rate, meaning we expect a sample size of at least N = 100 for the EFA, well within the minimum suggested sample size [37].

#### Data management

An extensive quality assurance and quality control system was developed by the study director for all study procedures, including recruitment, real time data checks, and random staff observations during all ethnographic activities during Aim 2. A database management system maintains and tracks all data including participant contact information, which is stripped of identifiers and stored on a password-protected computer with limited access to authorized staff.

### Ethical considerations

Offering easily accessible testing and then examining its correlates might obscure the “true” and likely lower prevalence of COVID testing uptake in a real-world setting. High rate of testing could challenge Aim 1’s main analyses of examining correlates and predictors of testing. However, healthcare access is typically infrequent in this population and barriers to service use are numerous. The study team felt that not offering COVID testing as part of the study—despite its potential to influence the primary outcome—would be unethical given the significant toll COVID has taken on the world’s population. Further, we hypothesize that even with accessible COVID testing, there will be variability in testing uptake owing to women’s perceived susceptibility to COVID or the anticipated impact of testing or COVID diagnosis on their lives.

### Study timeline and status

We began recruiting participants for Aim 1 and are presently still recruiting. With surging COVID case rates due to the Omicron variant, the study team paused in-person data collection activities in January 2022 for staff and participant safety. With no other interruptions in data collection, we anticipate completing recruitment in Summer 2023. In Winter 2022, we will begin Aim 2 activities including interviews with SPARC staff, observations of mobile outreach, and POCT implementation.

## Discussion

### Limitations and strengths

The study is characterized by several limitations. One limitation is that the target population is mobile and can be difficult to find for follow-up, even over a 6-month period. Building on the past decade of work with this population, we will continue to employ innovative and intensive strategies to locate WWUD [38]. Conservative assumptions in the power calculations also allow for losses to follow-up.

Numerous strengths characterize the study and its potential impact on the diffusion of COVID testing and vaccination research and practice. The CARE study population is important yet rarely the focus of tailored early prevention efforts for commonplace diseases such as influenza or COVID. Reducing the disease burden among this population will best occur through targeted interventions. Our proposed recruitment method is rigorous and will thereby enhance the study’s generalizability within Baltimore and other urban settings. The study’s relevance will further be increased by our commitment to hire staff who have lived experiences around drug use. Lastly, the study will provide important preliminary data and lay the groundwork for larger scale COVID testing and vaccine trials.

### Dissemination

At the end of the second year, we will hold several community meetings with partnering organizations such as SPARC to present study results to community members and local service providers. We will also distribute fact sheets about relevant study results to WWUD and relevant service organizations for distribution, as we have done in past studies.

The study fills a timely gap in the literature, given continued shortcomings in reaching WWUD in service engagement without the additional urgency of COVID’s impact. The study will directly inform the scale up of community-informed COVID testing that is accessible, low-barrier, and potentially serve as a bridge to utilization of other services. Our findings may also have implications for similar populations who do not access healthcare, are greatly impacted by reduction in health services during COVID, or for whom engaging in COVID prevention measures are challenging.
